# Vegan Red: A Safer Alternative to Synthetic Food Dyes?

**DOI:** 10.3390/toxics13060447

**Published:** 2025-05-28

**Authors:** Chiara Fogliano, Alessandra La Pietra, Chiara Maria Motta, Teresa Mobilio, Teresa Capriello, Margherita Sasso, Bice Avallone, Ida Ferrandino

**Affiliations:** Department of Biology, University of Naples Federico II, Via Cinthia 21, 80126 Naples, Italy; chiara.fogliano@unina.it (C.F.); alessandra.lapietra@unina.it (A.L.P.); mottacm@unina.it (C.M.M.); teresa.mobilio@unina.it (T.M.); teresa.capriello@unina.it (T.C.); marghe.sasso@gmail.com (M.S.); bice.avallone@unina.it (B.A.)

**Keywords:** zebrafish embryos, toxicity, vegan red, E120, E124, gene expression, muscle ultrastructure, behaviour

## Abstract

Food colourants are widely used additives classified as either synthetic or natural. In recent years, consumers have increasingly favoured natural options, considering them safer and potentially beneficial due to their nutritional properties. This study examined the effects of a natural food colourant, commercially known as Vegan Red (RVEG), on zebrafish embryonic development. Its impact was compared with cochineal red E120, of animal origin, and the synthetic dye E124, which are associated with hyperactivity in children and allergies. Shield stage embryos were exposed for 72 h and then examined using a multidisciplinary approach to assess the effects on conventional toxicity endpoints, such as survival, hatching rate, heart rate, genotoxicity, and behavioural interferences, including the impact on muscle ultrastructure. The results demonstrated that RVEG, as well as E120, do not affect hatching, heart rate, and motility parameters. However, RVEG moderately alters skeletal muscle organisation and, more relevant, the expression of the *gfap*, *chchd2,* and *notch1a* genes. Based on standard toxicity parameters, the findings indicated that RVEG is less toxic than E124 and E120, but that the alterations induced in gene expression and muscle anatomy raise safety concerns.

## 1. Introduction

Food dyes are common additives that make foods attractive to consumers [1]. Since the 1970s, their use has been subjected to rigorous evaluation by international organisations. The EFSA and WHO regularly re-evaluate the toxicity of various food additives, modifying their daily intake limits and, if appropriate, advising against or prohibiting their use to avoid harm to humans and the environment. As a result, international regulations are continuously updated, even if the directives are not implemented at the same pace by different nations. Consequently, the legislation varies from country to country, and the lack of uniformity poses a problem for food importers and exporters, as the same food colourant may be legal and illegal simultaneously.

Food colourants are classified as either synthetic or natural. The synthetic ones are cheap, more stable on shelves, and provide no nutritional value. They, however, have many adverse health effects. For example, Tartrazine Yellow E102 induces brain damage in mice and zebrafish [2] and is carcinogenic [3], while Ponceau 4R, also known as E124, induces hyperactivity and movement alterations in infants [4]. The information on the health effects of many other colours, such as Patent Blue E131 [5] and Allura Red E129 [6], remains controversial.

Consumers have paid greater attention to food safety in recent years, leading to the widespread use of natural colourants and, more recently, of vegan compounds derived from fruits and vegetables such as beetroots and red berries [7]. These products contain several bioactive molecules, including anthocyanins and phenols, which may significantly affect the physiology of organisms [8].

The safety of food colours available on the market remains to be established. Although they are added to food in small quantities, they are present in a wide variety of daily-used foods and beverages, even when not strictly necessary. Indeed, in the case of natural compounds such as vegan food dyes, their consumption is practically unlimited since they are considered safe and health beneficial.

Their intensive use leads to the release of food dyes into the environment, especially into water bodies and irrigated land, with potential consequences for ecosystems. Little is known about the effects of food dyes, particularly natural ones, on organisms, so defining their toxicity is essential. Abe et al. [9] demonstrated that synthetic and natural dyes affect freshwater organisms. Other studies have highlighted the effects of different food dyes on plants [10,11,12], adults and larval invertebrates [13,14] and vertebrates [10,15]. In particular, three red dyes, E124, E120, and a vegan red (RVEG), showed adverse effects on *Cucumis sativus*, *Artemia salina*, and cultured human cells (PNT1A) [10,11]. In *Cucumis sativus* and *Artemia salina,* toxicity has been linked to the onset of oxidative stress, but not in *Danio rerio* [12].

Therefore, the present work aimed to test the effects of vegan red dye on the embryonic development and motility of *Danio rerio* (zebrafish) and to compare it to the other two food colours, E120 and E124. The tests were conducted under the same conditions described in Motta et al. [10], and toxicity parameters were first assessed to determine food dye effects. Larval motility parameters were also evaluated, namely spontaneous movements within the chorion and, after hatching, larval velocity, acceleration, distance moved, and number of movements. All the tests employed are validated in zebrafish, an excellent model for motility and neuro-behavioural studies [16,17]. Parallel real-time PCR investigations assessed the expression of *gfap*, a marker of neurotoxicity expressed in astrocytes and glial cells [18,19], *chchd2*, which is implicated in apoptosis and mitochondrial respiration and stress [20], and *notch1a*, which is involved in notch signalling and cardiogenesis, and expressed in the nervous system, spinal cord, and tail [21,22,23]. Finally, transmission electron microscopy investigations were conducted to verify muscle integrity and to correlate this with changes in behaviour.

## 2. Materials and Methods

### 2.1. Preparation of Red Food Dye Solutions

Three food dyes, commonly available on shelves and widely used in home food preparation, were selected for this study. According to the manufacturer, the vegan red dye, sold in powder form, contains extracts of radish, black currant, and apple in unspecified proportions. Ponceau Red 4R (E124) and Cochineal Red (E120) were supplied in 3 mL vials, each containing water and 20% dye. While there are no legal restrictions on these natural dyes, manufacturers have complete discretion over the type and concentration of colourants used. All experiments used the same product brand and batch to minimise variability. The dyes were diluted in mineral water (pH 7.4, salinity 357 mg/L) to a final concentration of 1.2 g/L for treatment [10,11].

### 2.2. Embryo Growth

Embryos were generated from healthy adult zebrafish housed in oxygenated tanks with a natural photoperiod of 14 h:10 h light/dark, at a temperature of 28.0 °C and a pH of 7.5 [24]. Fish were fed twice daily with a commercial diet supplemented with *Artemia* sp. nauplii [25]. Eggs, spawned at the first morning light, were collected with a syphon from the fish tanks and housed in dishes containing E3 medium (5 mM NaCl, 0.17 mM KCl, 0.33 mM CaCl_2_·2H_2_O, 0.33 mM MgSO_4_) in a water bath at 28.0 °C [25]. Embryos were selected for experimental procedures at 6 h post fertilisation (hpf). All experiments were conducted according to the Italian (D. lgs 26/2014) and European (2010/63/EU) guidelines on the welfare of animals used for research purposes.

### 2.3. Embryo Treatment

Embryos at the shield stage were randomly assigned to plates containing 10 mL of the respective treatment solutions or mineral water for the control group [10] and left to develop for 72 h. The treatments were conducted in six-well plates, with 10 embryos per well, in duplicate (20 embryos for each group). The embryos were incubated at 28.0 °C in 10 mL of solution, renewed daily, for 72 h. Each treatment was repeated in triplicate.

### 2.4. Hatching and Heart Rate Analyses

The hatching rate was determined by counting the number of hatched larvae against the total number of developing embryos, at 24 h, 48 h, and 72 h of treatment [24].

The heart rate was determined by placing the larvae, treated for 72 h, in a drop slide and counting the beats under a light microscope. Three replicate measures of 15 s were obtained, and the average was used to calculate beats per minute [26].

### 2.5. Behavioural Analyses

#### 2.5.1. Spontaneous Embryonic Movements

Spontaneous movements were assessed in embryos after 24 h of exposure. Three embryos per group were placed in a drop slide and examined for 1 min under a light microscope. Observations were repeated three times. Data were expressed as the average number of movements per minute (n/min).

#### 2.5.2. Motility Test at 72 h

Six larvae were randomly selected from the treated and control groups and individually transferred to 3.5 cm diameter Petri dishes. Care was taken to minimise handling time and avoid physical damage, noise, and rapid movements to reduce stress. After 10 min of acclimatisation, larval motility was recorded for 5 min [27]. The recorded videos were analysed using Tracker 4.84 Video Analysis and Modeling Tool at five-frame intervals (0.5 s). The following parameters were evaluated: velocity (cm/s), acceleration (cm/s^2^), total distance moved (cm), and the number of movements per five-frame interval, which reflects larval activity during the observation period [15].

### 2.6. Histological Analyses

Ten randomly selected larvae per treatment were fixed in 2.5% glutaraldehyde + 4% paraformaldehyde (24 h, at 4 °C) and post fixed in 1% osmium tetroxide (1 h, at 4 °C). After a series of washes in 0.1 M PBS pH 7.4 at 4 °C to remove fixative residues, the larvae were dehydrated in ascending ethanol, pre-infiltrated in propylene oxide-Epon 812, and embedded in Epon 812 (60 °C, 48 h) [28].

Semi-thin sections (1.5 μm) of control and treated tails were prepared with a glass knife and stained with 1% toluidine blue. For each treatment, thirty serial sections were examined and photographed with a Zeiss Axiocam camera applied to a Zeiss Axioskop microscope (Zeiss, Jena, Germany).

Ultrathin sections (50–80 nm) were cut with a diamond knife at an angle of 45 degrees (Diatome, Biel, Switzerland) and loaded on 200-mesh grids. After staining with 3% uranyl acetate in 50% ethyl alcohol and 2.6% lead citrate, the sections were observed in a FEI Tecnai G2 electron microscope (Hillsboro, Oregon) at 120 kV.

### 2.7. Quantitative Real-Time PCR

The expression of the *gfap*, *chchd2,* and *notch1a* genes was analysed by quantitative Real-Time PCR (qRT-PCR) as previously described [29]. Twenty larvae per group were homogenised, and RNA was extracted using Direct-zol^TM^ RNA Miniprep Plus Kit (ZYMO RESEARCH, Irvine, CA, USA). Then, the Nanodrop^®^ spectrophotometer 2000 (Thermo Scientific Inc., Waltham, MA, USA) measured the concentration and purity of RNA. A cDNA strand was synthesised from 1000 ng of the total RNA using an All-In-One 5X RT MasterMix (Applied Biological Materials, Richmond, BC, Canada). For qRT-PCR, a reaction was performed using 2 μL of cDNA and 0.5 μL of each primer (Table 1) (Eurofins Genomics, Ebersberg, Germany) at a concentration of 10.0 μM, with the BlastTaqTM 2X qPCR MasterMix (Applied Biological Materials, Richmond, BC, Canada). The thermocycling conditions were 1 cycle for enzyme activation (95 °C for 3 min), followed by 40 cycles for denaturation and annealing/extension (95 °C for 15 s, 60 °C for 1 min), and a melting curve analysis performed according to the instructions provided with the StepOnePlus Real-Time PCR system (Thermo Fisher Scientific Inc., Waltham, MA, USA). The *β-actin* gene was used to normalise the expression of each gene through Ct value using the REST software (Relative Expression Software Tool, version 1.9.12) according to Pfaffl’s method [30,31].

### 2.8. Statistical Analysis

All experiments were conducted in triplicate. GraphPad Prism 7 (GraphPad Software, La Jolla, CA, USA) was used to assess statistically significant treatment differences using one-way ANOVA followed by Tukey’s pairwise comparison tests. Data expressed as mean ± SEM were considered statistically significant if *p* < 0.05.

## 3. Results

### 3.1. Toxicity Parameters

**Figure 1:**
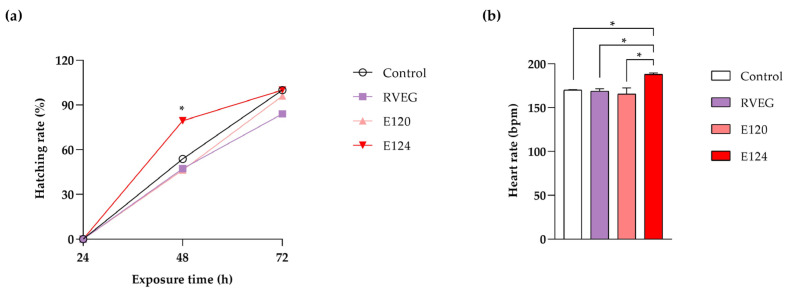
Effects of red dyes on hatching rate at 24, 48, and 72 h of exposure (**a**) and heart rate at 72 h of exposure (**b**). Significant differences were determined using one-way ANOVA followed by Tukey’s test (* *p* < 0.05).

The acute toxicity test did not reveal significant mortality events in the treated groups compared with the control throughout the treatment period. Regarding hatching, a significantly increased rate (*p* < 0.05) was observed at 48 h of exposure in the group treated with E124 compared with the control and other treated groups (Figure 1a). The heart rate, measured at 72 h of exposure, increased significantly (*p* < 0.05) in E124-treated animals compared with the control and other treated groups (Figure 1b).

### 3.2. Behavioural Tests

#### 3.2.1. Assessment of Spontaneous Embryonic Movements

**Figure 2:**
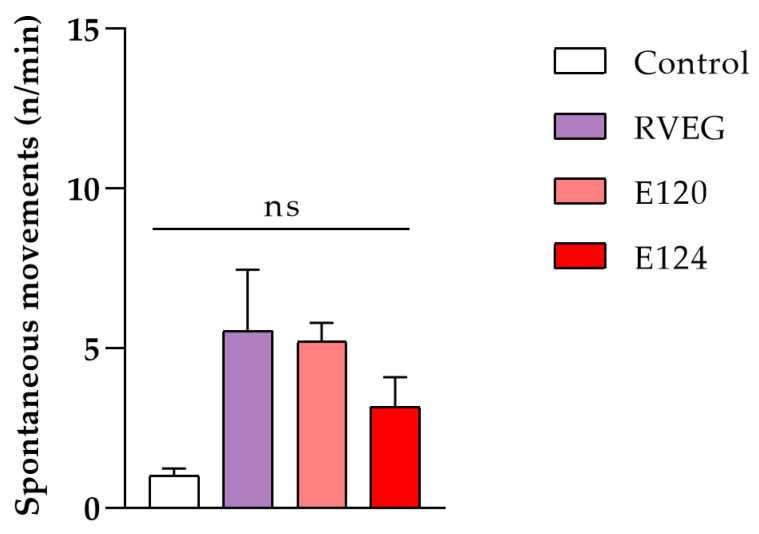
Effects of red dyes on the embryos’ spontaneous movements while in the chorion. No significant differences were noticed (ns = not significant; one-way ANOVA followed by Tukey’s test).

After 24 h of exposure, spontaneous movements increased in RVEG (Figure 2), although the increase was not statistically significant (*p* > 0.05).

#### 3.2.2. Evaluation of Swimming Performance

**Figure 3:**
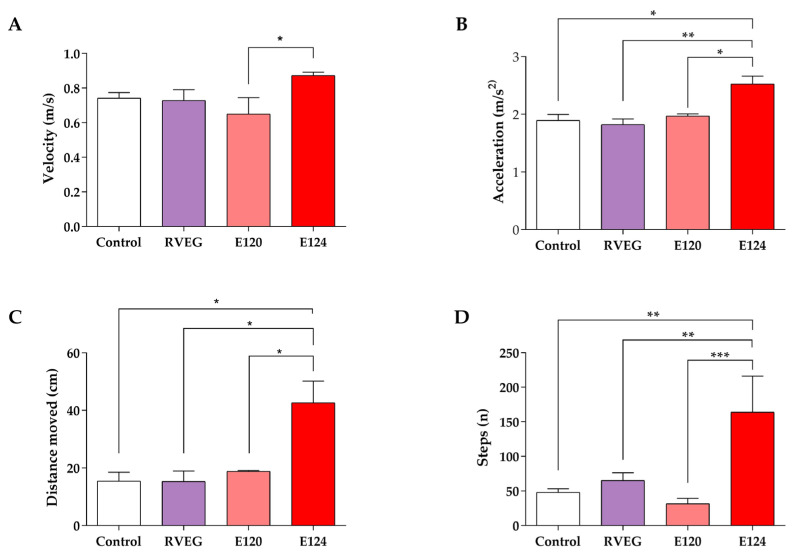
Motility parameter of *Danio rerio* larvae, control or exposed to vegan red (RVEG), E120, or E124 for 72 h. (**A**) Velocity test (cm/s). (**B**) Acceleration test (cm/s^2^). (**C**) Distance moved (cm). (**D**) Steps (n). One-way ANOVA followed by Tukey’s test (* *p* < 0.05; ** *p* < 0.01; *** *p* < 0.001).

The average velocity (Figure 3A) and acceleration (Figure 3B) did not change significantly in RVEG and E120 larvae compared with the control (*p* > 0.05). In contrast, in line with reports on hyperactivity, E124-treated larvae showed a marked increase in both velocity and acceleration (*p* < 0.05). Specifically, the velocity rose from 0.74 ± 0.01 cm/s in controls to 0.87 ± 0.01 cm/s in E124-treated larvae, while the acceleration increased from 1.89 ± 0.04 cm/s to 2.52 ± 0.07 cm/s in E124 samples.

No effects were observed on the distance moved (Figure 3C) and steps (Figure 3D) in RVEG and E120 larvae with respect to the controls (*p* > 0.05). In contrast, the larvae exposed to E124 travelled 37 ± 4.13 cm, 2.4 times farther than the controls (15.45 ± 1.25 cm). This result, in line with the 3.5-fold increase in steps (*p* < 0.01), further confirms that E124 induces hyperactivity in fish.

### 3.3. Histological Analysis of Skeletal Muscle

**Figure 4:**
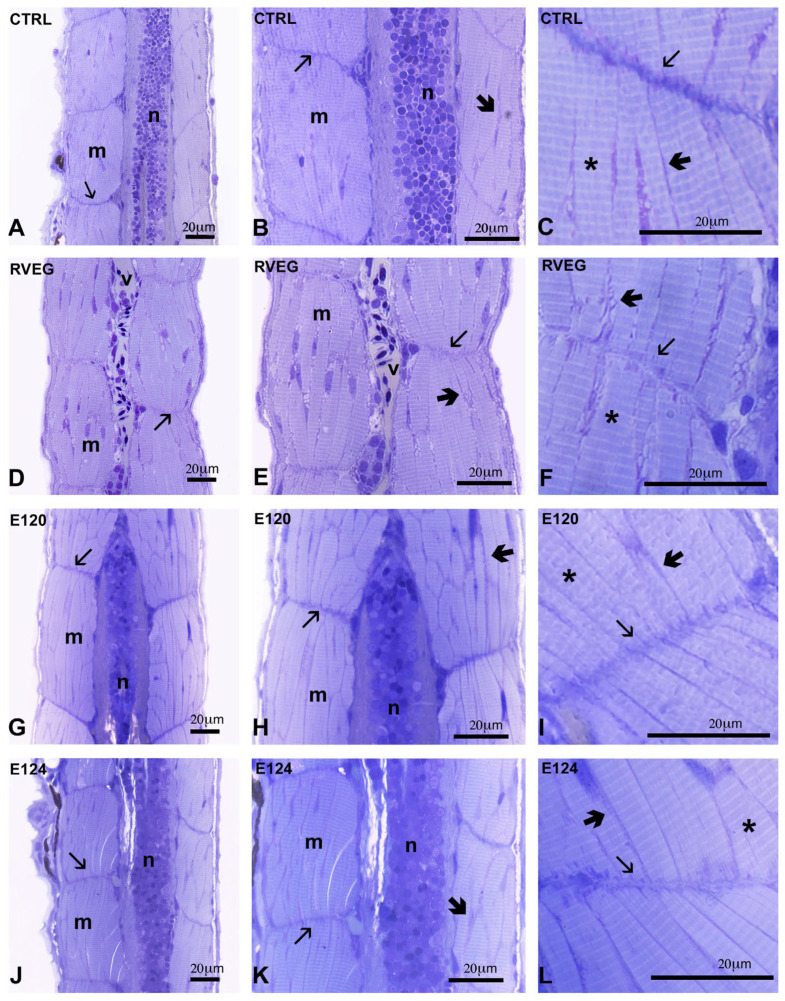
Skeletal muscle organisation in *Danio rerio* larvae, control or exposed to vegan red (RVEG), E120, or E124 for 72 h. (**A**–**C**) In control groups, dense myosepta (thin arrows) separate myomeres (m) containing regularly organised fibrils (*). Notice scarce interfibre connectives (thick arrows). (**D**–**F**) RVEG group displayed poorly stained myosepta (thin arrows), regular banding (*), and scarce interfibre connectives (thick arrows). In E120 (**G**–**I**) and E124 (**J**–**L**) groups, dispersed and pale myosepta (thin arrows), thick connectives separate single fibres (thick arrows), and regular banding (*) are visible. Myomers (m), neural tube (n), tail vessel (v). Semi-thin sections stained with toluidine blue. Magnification: 20×; 40×; 100×.

In the control larvae, the muscle fibres were regularly organised, showed the typical banding, and were separated by thin endomysial connectives containing the eccentric nuclei (Figure 4A–C). The myosepta appeared dense, regularly thick, and typically undulated by the presence of sarcolemma regular invaginations.

In the RVEG larvae (Figure 4D,F), the myosepta appeared loose and poorly stained, the endomysial connectives were scarce (Figure 4E), while fibre banding (Figure 4F) was regular. In the E120 and E124 larvae, the myosepta were dispersed and pale (Figure 4I,L) and the connectives were particularly abundant, clearly lining each fibre (Figure 4G,H,J,K). The banding was regular (Figure 4I,L).

### 3.4. Ultrastructure of Skeletal Muscle

**Figure 5:**
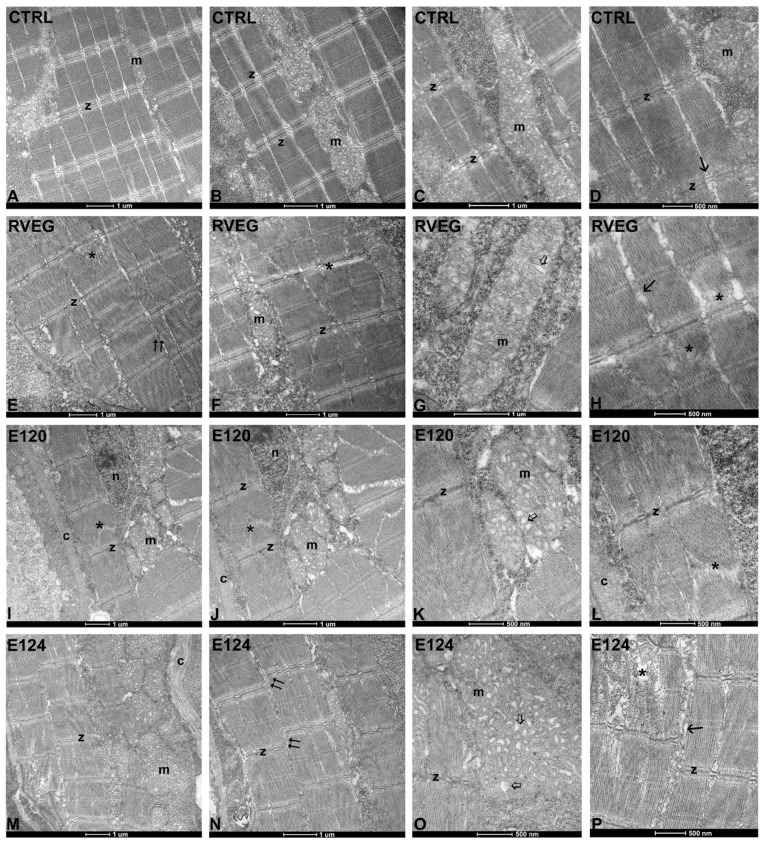
Ultrastructure of skeletal muscle in *Danio rerio* larvae, control or exposed to vegan red (RVEG), E120, or E124 for 72 h. (**A**–**D**) Regularly organised myofibrils with evident banding and Z-bands (z) in register are visible in the control group. Mitochondria (m) with tubular cristae and sarcoplasmic reticulum (arrow). (**E**–**P**) In dye-treated larvae, irregularly disposed myofibrils (*) with Z-bands (z) that are not always in register (double arrows) are visible. Dilated mitochondrial (m) cristae (empty arrows) and increased sarcoplasm among myofibrils (arrows). Significant increase in collagen content (c). Nuclei (n).

The control muscle (Figure 5A,B) exhibited regularly organised fibres, with sarcomeric myofibrils forming the typical banding (Figure 5A,D). The Z-bands were aligned in register. The sarcoplasmic reticulum neatly separated the myofibrils (Figure 5D). The mitochondria were numerous, with a regular shape and dense tubular crests (Figure 5C).

Significant alterations were observed in the dye-treated larvae. In particular, the fibres were slightly out of register after exposure to RVEG (Figure 5E) and firmly disaligned in E124 (Figure 5N). In addition, in all treatments, sarcomeres showed disorganised myofibrils (Figure 5E,I,J), particularly after exposure to E124 (Figure 5P). The mitochondria showed disorganised vesicular cristae in a mild form in RVEG (Figure 5G), and more severely in E120 and E124 (Figure 5K,O). E120 (Figure 5I,J) and E124 (Figure 5M,N) showed a significant increase in collagen content.

### 3.5. Analysis of Gene Expression

**Figure 6:**
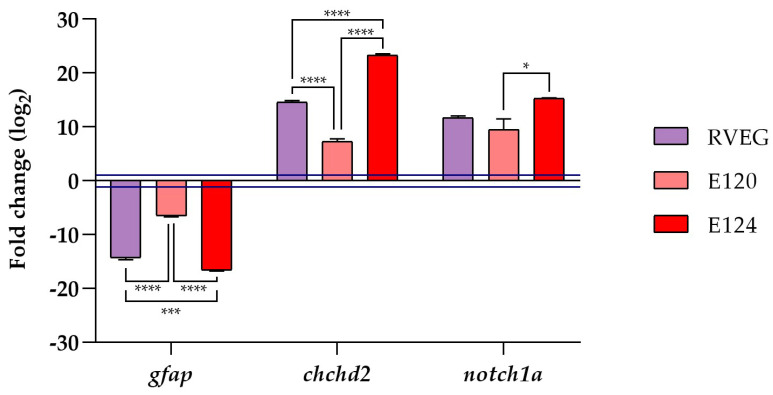
Effects of red food dyes on *gfap*, *chchd2,* and *notch1a* expression. Fold changes were calculated as: fold change = 2^−ΔΔCt^. Blue lines indicated the fold change thresholds of 2 and 0.5, respectively. Values above 2 and below 0.5 were considered significant compared with the control. One-way ANOVA followed by Tukey’s test (* *p* < 0.05; *** *p* < 0.001; **** *p* < 0.0001).

The gene expression analysis was conducted through qRT-PCR at 72 h of exposure. The results (Figure 6) showed a downregulation of *gfap* in all treated groups compared with the control. In contrast, *chchd2* and *notch1a* were upregulated in all groups exposed to the three food dyes.

## 4. Discussion

In this work, a comparison was made between RVEG and the two red dyes E120 and E124 in *Danio rerio* during the early stages of *Danio rerio* embryo development. The data showed that RVEG does not affect hatching and behaviour, a result supported by the unchanged heart rate, a biomarker of stress [32]. This result aligns with previous reports in *Danio rerio* exposed to other dyes, such as sunset yellow [33], but disagrees with evidence obtained with E150d [15] or with red dyes in another aquatic model, *Artemia salina* nauplii. In this invertebrate, the dye decreases hatching and slows growth [11]. The different responses in the two species may depend on the differences in development, the innate antioxidant system, and/or the pro- and anti-oxidant properties of polyphenols [34].

The data obtained on E124 confirm previous experiments [10], demonstrating that it increases the heartbeat rate and stimulates hatching in *Danio rerio* larvae. Both effects can be correlated with the induction of oxidative stress [14], although the exact mechanism of action remains to be clarified, as the collected data are contradictory. E124, by inducing cardiac oedema [10], should cause bradycardia [15,35], not tachycardia, and should delay, not accelerate, hatching, having anti-proliferative effects [36,37]. However, the data in *Danio rerio* are confirmed by similar results in *Artemia salina* nauplii [11], indicating that further investigations are needed.

More consistent with the literature is the E124-induced larval hyperactivity. The absence of any effects in pre-hatched embryos can be explained by considering the immaturity of the motoneuronal system, which is fully functional only five days post fertilisation [38].

The tests in *Danio rerio* also confirm that E120 is safe [39] and does not interfere with embryo and larval development. It is not teratogenic in *Danio rerio* [10] and does not affect hatching, heart rate, or larval motility before or after hatching. The allergic reactions reported in humans are excluded in larvae, where the immune system becomes morphologically and functionally mature 4 to 6 weeks post fertilisation [40]. E120, however, is a pro-oxidant, inducing ROS via degradation to aromatic amines [41]. The apparent absence of effects can be attributed to the high efficiency of the innate antioxidant system of *Danio rerio* larvae [12,42].

The lack of effects of E120 and RVEG on larval mobility agrees with the lack of impact on in vivo routine oxygen consumption and ROS levels [12]. These results suggest that no change would occur in non-mitochondrial respiration, which is maintained at high levels during early embryogenesis to prevent oxidative stress [43]. Two intriguing pieces of evidence question the safety of the two natural red dyes on larvae. The first is a significant decrease in *in vitro* susceptibility to oxidative stress [12]. The second is the twofold increase in spontaneous movements registered in the pre-hatched larvae exposed to RVEG. Though not statistically significant, this latter result prompted further investigation.

Microscopy analyses indicate that the three red dyes affect muscle organisation differently, with E124 > E120 > RVEG. RVEG affected the transverse myosepta, which appeared loose and poorly stained by toluidine blue. Collagen fibrils should be deposited at hatching in a dense and compact structure indented with the sarcolemma [44]. The evidence, however, suggests that the myosepta were disorganised, which would indicate impaired force transmission during contraction. Reduced motor performances, however, were not detected, and further investigation is needed to clarify what happens in later stages.

E120 and E124 increased the amounts of connectives present among fibres, a factor that reduces muscle quality [45]. Although the intramuscular connectives vary enormously in functionally different muscles, they always constitute a scaffold essential for fibre development and growth, carrying blood vessels and nerves to the muscle cells. Therefore, the observed variations in this component point to a significant decrease in muscle mechanical properties. In addition, the concomitant reduction in *gfap* expression indicates the improper development of vascular smooth muscle and endothelial cells [46], further pointing to reduced motor efficiency. Performance remained unchanged in the larvae, but no evidence is available on what happens in adults.

The evidence that the three dyes induce fibril mispositioning and sarcomere misalignment is also very relevant. The myofibrillar apparatus is modestly altered, which is probably insufficient to reduce contraction efficiency. The absence of nuclear relocation, a phenomenon observed in several forms of muscle atrophy [47], supports this conclusion. The misalignment of sarcomeres suggests an interference with desmin, the intermediate filaments that surround and connect the sarcomeres in the Z-line [48]. Reduced alignment is also associated with delayed fibre maturation [49], a process that depends on a timed temporal switch from *Notch* to *Wnt* signalling [50,51]. The overexpression of Notch1a observed in *Danio rerio* larvae supports the altered myoblast differentiation.

Another evident alteration was observed in mitochondria, where the altered cristae indicate an interference with the metabolism and redox homeostasis [52]. Energy deficiency should be excluded, as no changes were observed for in vivo routine oxygen consumption [12] or in larval motility. Maintaining a full motor capacity in *Danio rerio* larvae would depend on the *chchd2* overexpression. The gene encodes for a small mitochondrial protein that resides in the intermembrane space and plays a crucial protective role in the case of mitochondrial dysfunction [53].

Overall, none of the three red dyes tested proved utterly safe, which is somewhat surprising for the RVEG product due to its vegetal origin. It is well known that red fruit preparations contain high amounts of biologically active substances. Some of these raise no concern since no adverse effects have been described. For example, betalains (betacyanins and betaxanthins), present in beetroots, a common component of red food dyes, have marked positive effects and are used by athletes to favour muscle recovery [54]. The role of polyphenols is more complex, as they can have both positive and adverse effects, being anti- and pro-oxidant [34]. Other principles are non-beneficial or even toxic. Oxalic acid, for example, is a natural antioxidant [55] and a potent iron and calcium chelator. High concentrations (several mg/L) cause severe interference with the metabolism and, in aquatic organisms, potential respiratory distress, osmoregulatory imbalance, gill damage, and neurological symptoms [56].

## 5. Conclusions

In conclusion, the food additive RVEG is safer than E120 and E124. However, its use should be treated with caution, especially in children, considering the effects it exerts on gene expression in *Danio rerio* at 72 h of exposure to this colourant. This evidence also highlights a potential environmental risk that has so far been underestimated. Vegan products are widely consumed, but their presence in the environment is wholly ignored; the beneficial factors driving their use may potentially interfere with a plethora of non-target organisms.

## Figures and Tables

**Table 1:** Primer sequences of genes used for qRT-PCR. F (forward); R (reverse).

Gene	Symbol	Primers		Accession No.
Actin1	*β-actin*	F	CGAGCAGGAGATGGGAACC	NM_131031.1
		R	CAACGGAAACGCTCATTGC	
Glial fibrillary acidic protein	*gfap*	F	GCTCTGAGACAAGCGAAGCA	NM_131373.2
		R	CACGGAGAGATTCCAGGTTCA	
Coiled-coil-helix-coiled-coil-helix domain	*chchd2*	F	TGTCGGACACACAATAGGTCAC	NM_200767.1
		R	CATATGAACATGGGTTCTGCTG	
Notch receptor 1a	*notch1a*	F	ACATCACCCTTCCAGCAGTC	NM_131441.1
		R	AGGCTTCCCTAAACCCTGAA	

## Data Availability

Data are available from the corresponding author upon reasonable request.
